# Germination fitness of two temperate epiphytic ferns shifts under increasing temperatures and forest fragmentation

**DOI:** 10.1371/journal.pone.0197110

**Published:** 2018-05-11

**Authors:** Jose María Gabriel y Galán, Antonio Murciano, Laure Sirvent, Abel Sánchez, James E. Watkins

**Affiliations:** 1 Unit of Botany, Department of Biodiversity, Ecology and Evolution, Faculty of Biology, Universidad Complutense, Madrid, Spain; 2 “Neural Plasticity Research Group, IdISSC”, Unit of Biomathematics, Department of Biodiversity, Ecology and Evolution, Faculty of Biology, Universidad Complutense, Madrid, Spain; 3 “Neuro-computing and Neuro-robotics Research Group, UCM”, Unit of Biomathematics, Department of Biodiversity, Ecology and Evolution, Faculty of Biology, Universidad Complutense, Madrid, Spain; 4 National Museum of Natural History, Paris, France; 5 Department of Biology, Colgate University, Hamilton, New York, United States of America; Fred Hutchinson Cancer Research Center, UNITED STATES

## Abstract

Ferns are an important component of ecosystems around the world. Studies of the impacts that global changes may have on ferns are scarce, yet emerging studies indicate that some species may be particularly sensitive to climate change. The lack of research in this subject is much more aggravated in the case of epiphytes, and especially those that live under temperate climates. A mathematical model was developed for two temperate epiphytic ferns in order to predict potential impacts on spore germination kinetics, in response to different scenarios of global change, coming from increasing temperature and forest fragmentation. Our results show that an increasing temperature will have a negative impact over the populations of these temperate epiphytic ferns. Under unfragmented forests the germination percentage was comparatively less influenced than in fragmented patches. This study highlight that, in the long term, populations of the studied epiphytic temperate ferns may decline due to climate change. Overall, epiphytic fern communities will suffer changes in diversity, richness and dominance. Our study draws attention to the role of ferns in epiphytic communities of temperate forests, emphasizing the importance of considering these plants in any conservation strategy, specifically forest conservation. From a methodological point of view, the model we propose could be easily used to dynamically monitor the status of ecosystems, allowing the quick prediction of possible future scenarios, which is a crucial issue in biodiversity conservation decision-making.

## Introduction

Global warming and changes in land use, derived from human activities, are shifting abiotic factors across multiple ecosystems around the world. Both models and empirical observations predict that such shifts could cause, among other effects, increased species extinctions, unexpected alterations of geographic range distributions, and disruption of ecosystem function [1–3]. Several studies on plants have already demonstrated organismal responses in many parts of the world including loss of forest structure and size [4, 5], shifts in species distribution [6], changes in phenology, development and physiology [7, 8].

At the population ecology scale, germination is an important biological process, as it directly influences population establishment and dynamics, thus setting the limits for plant distributions, species diversity and community composition [9]. Seed and spore germination requires a combination of suitable abiotic factors (temperature and moisture, among others), any alteration of which could significantly impact the process, posing serious consequences on populations and communities. In general, previous research reported that increasing temperatures affects germination, inhibiting, delaying or enhancing it [10–13]. Also, forest fragmentation has been negatively related to seed germination and offspring production [14], although seeds of some species seem to germinate independently of fragmentation [15]. In some cases, changes in germination have been attributed to changes in microclimate due to forest modification [16].

Unlike plants that are rooted in the terrestrial substrate, epiphytes have no connection to the ground or their host plants, fact that face them to a challenging environment considered in general as xeric [17, 18]. Given all the conditions of this ecological niche, it is easily understood that epiphytes, both vascular and non-vascular, respond rapidly to even minor changes in their environment, even though there exist differences in water uptake between these two functional groups [19–21]. The potentially higher vulnerability of epiphytes makes them worthy of special attention, and also promotes them as ideal models to study and predict the effects of global change on ecosystems [22, 23]. Regarding germination, epiphytes are affected by increasing temperatures due to global warming [17, 19, 21]. Forest structure related to fragmentation is also linked to epiphytic abundance as well-structured complex forests generate more buffered micro-conditions which maintain suitability for the epiphytic habit [20, 22, 24, 25]. The loss of forest structure due to fragmentation and clearness of the canopy affects, at least, epiphytic community composition by reducing individuals of intolerant species and promoting new suitable opportunities to tolerant ones [19, 26]. The anthropogenic effects of forest perturbation over epiphytes is complex and exhibit many subtle impacts [21], so much more future attention is needed in this subject.

Ferns are an important component of ecosystems around the world and can play important roles in ecosystem structure and function, particularly in the rainforest canopy where they can often be the most diverse and abundant members of the epiphytic community [27–30]. Ferns are also common in forests of temperate regions, where they can appear as epiphytes if their critical environmental requirements are met [31–33]. Despite this, studies of the impacts that global changes may have on ferns are scarce, yet emerging studies indicate that some species may be particularly sensitive to climate change [34–37]. The lack of research in this subject is much more aggravated in the case of epiphytes [21, 38–40], and especially those that live under temperate climates, which have been hardly studied [41]. This information points out that the global change is impacting the communities of epiphytic ferns, reporting data of expected changes in composition and abundances of species [35, 41], sometimes highlighting the importance of altitudinal gradients [42]. Nevertheless, all this work has been completed on the sporophytic phase. Thus, no details are offered on how global change is affecting germination of spores, which is a critical biological process in the fern life history [36], or over spore viability, trait that permits the maintenance of spore banks to ensure the presence of gametophytic populations [43].

The main aim of this study was to evaluate whether some aspects of the global change (increasing temperatures and loss of habitat due to forest fragmentation) influence the germination of temperate epiphytic fern spores. In this context, we wanted to test the following two hypotheses: a) spore germination will be modified under increasing temperature; and b) germination will be changed in populations established in fragmented forests. Also, we wanted to provide a mathematical model of the vulnerability of temperate epiphytic ferns, using spore germination as a functional trait related to population recruitment, to predict impacts under different scenarios of increasing temperature. In this study two main variables related to germination were measured to explore the impacts of climate change on temperate epiphytic ferns. First, a species’ maximum germination percentage, and second the delay in the onset of germination [44]. This model may be a valuable tool in management and decision-making regarding biodiversity conservation policies.

## Material and methods

### Biological material and sampling locations

**Fig 1:**
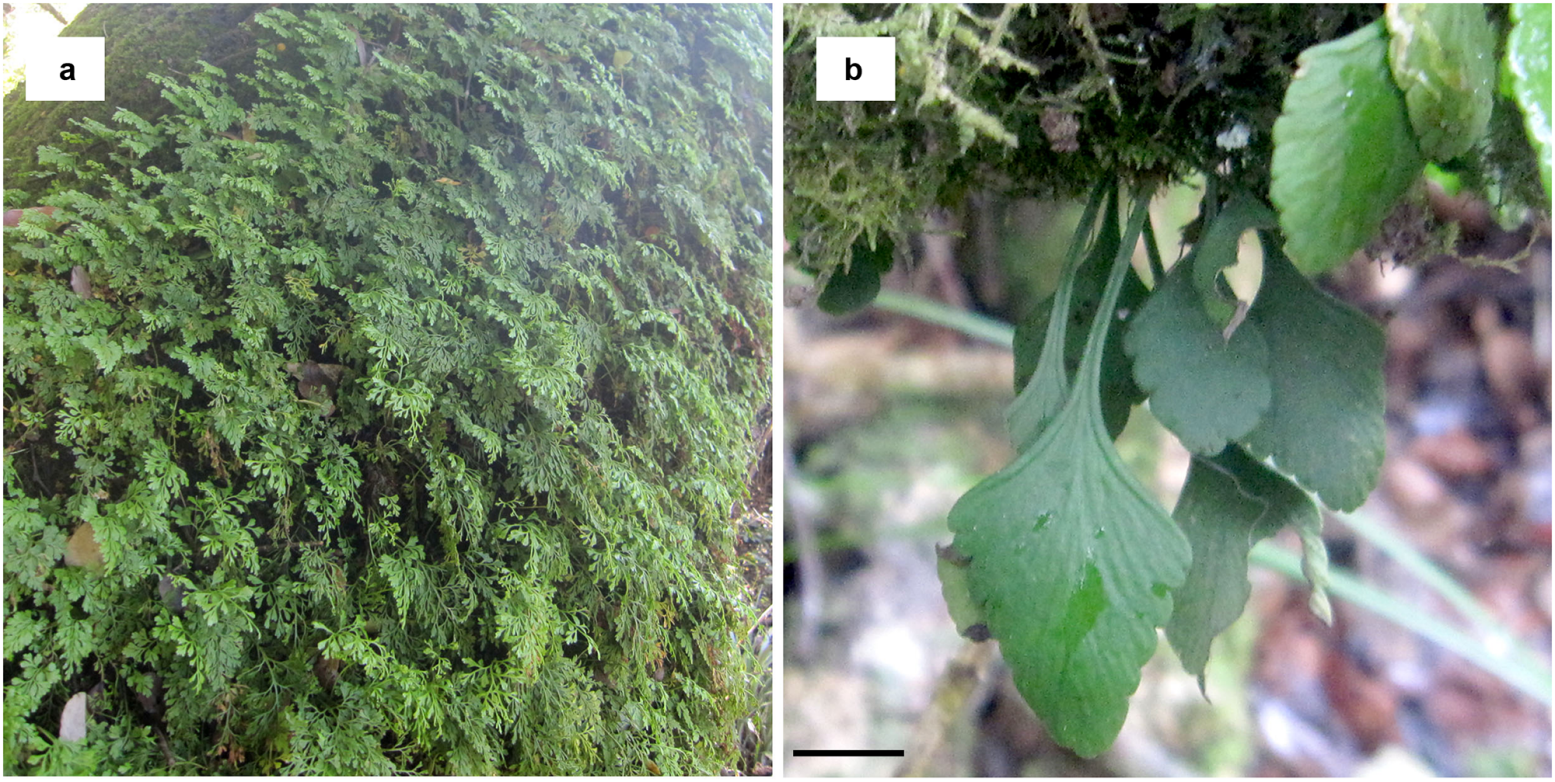
Species tested in this study. A: *Asplenium dareoides*; B: *Asplenium trilobum*. Bar = 12 cm in a, 1.5 cm in b.

For this study, we choose two epiphytic ferns *Asplenium dareoides* Desv. and *Asplenium trilobum* Cav. (Fig 1) that occurs in temperate forests of Chile, as members of the epiphytic communities on a wide variety of host trees. While *A*. *dareoides* is a moderately common plant in both Chile and Argentina (ranging about 31–55°S), *A*. *trilobum* lives mainly in Chile, with some scarce populations in Argentina, and has a more restricted distribution, about 36–46°S [45].

To test the influence of forest fragmentation, we collected material from two different populations of each species, separated for more than 400 km, and which occur on host forests with different level of deterioration. The extent of the fragmentation was determined by previous reports [5, 46, 47]. The first population (P1) was located in Chile, Región del Bío-Bío, Provincia de Concepción (36°47’S 73°09’W, 35 m), in a patch of quite altered forest with high values of anthropogenic fragmentation P_fa_ and low values of forest connectivity P_ff_ [5]. The second population (P2) was located in Chile, Región de los Lagos, Provincia de Osorno (40°40’S 72°09’W, 465 m), within a large area of well-structured primary forest, showing very low values of anthropogenic and natural fragmentation P_fa_ and P_fn_, respectively, and high values of forest connectivity P_ff_ [5].

At each location, we collected 5 different sporophytes of each species, growing on 5 different trees, separated at least 80 m each other. The total number of individuals per species were 10, 5 per population. These species were identified using a local flora [45]. Collections in both locations were done in collaboration with the Universidad of Concepción (Chile): locality 1 is included in a biological research station owned by the University itself and sampling did not require special permission; for the sampling of locality 2 the university had a permission issued by the Puyehue National Park Directorate. In any case, none of the species used in this study is endangered or particularly threatened. One herbarium voucher per species was deposited in the herbarium MACB (*A*. *dareoides* MACB 108700, *A*. *trilobum* MACB 108701).

### Germination experiments

Fertile fronds from each individual were separately placed in sealed envelopes and spores were allowed to dehisce for 24 hours under a source of moderate heat. Spores were sown in mineral agar [48] and cultured in Petri plates in a germination chamber. As our interest here was to test the influence of increasing temperatures over germination, the rest of environmental parameters were maintained constant, at more or less standardized conditions for fern spores culture [44, 48]: no-depletion mineral nutrition, culture density of about 20 spores/cm^2^, saturated humidity within the sealed plate, 12-h light photoperiod and constant light regimen from daylight fluorescent tubes at a photon irradiance of 30–45 μmol m^-2^ s^-1^. These factors are known to influence fern spore germination and gametophyte development, and future experiments should be addressed to globally understand the response of epiphytic fern germination to global change considering each of these aspects.

Regarding temperature, we disregarded the use of extreme maximum and minimum temperatures as control values, due to the known limiting effects on most of the biological processes, and specifically over fern spore germination [44, 49]. Assuming that further investigation is needed to assess the effects of daily/annual temperature fluctuations, we decided to use a temperature of 13°C as representative of the mean annual temperature of both locations, which are 12.9°C for P1 and 12.6°C for P2 [50]. A temperature of 17.5°C was selected as a temperature representing a scenario RCP8.5 of increasing temperature (+4.5°C) [51].

Each experiment (i.e., each species/individual/location/temperature) was replicated three times, for a total of 120 culture plates. Every three days, plates were observed under a compound microscope and proportion of germinated spores was counted looking at 100 spores randomly selected. Spores were marked as germinated when a rhizoid was evident [44]. The observation period for each plate concluded when there was no increase in the germination percentage for two consecutive observations (ranging from 33 days for the quicker experiment, to 39 days for the slower plate).

Two main variables have been measured to explore possible changes in germination kinetics due to climate change: first, the maximum percentage of germination reached (G); and second, the delay in the onset of germination (D), measured in days (this variable represents the delay in days between theoretical and experimental curves if germination velocity is maximum from the beginnings). Both variables are related to environmental factors but they have also biological meaning, regarding spore age and other endogenous features [44].

### Statistical procedures

First, germination data over time from each scenario (i.e. species, location and temperature) was fit to a sigmoidal growth curve of the type:

$$y(t)=\frac{G}{1+e^{\frac{4\mu (D-t)}{G}+2}}$$

where y(t) represents the mean proportion of germination at t time, G the carrying capacity (i.e. the maximum proportion of germination), μ the maximum growth rate (i.e. the derivative at the inflection point of the curve) and D the delay time. Fitting was done by means of non-linear regression, so coefficients were estimated by an iterative least squares estimation using Marquardt method (maximum number of iterations set to 200, termination tolerance on estimated coefficients set to 10^−12^ and termination tolerance on residual sum of squares set to 10^−12^).

Then, we analyzed G and D, for each species / population / temperature, through a Generalized Estimating Equation (GEE) procedure, including these factors and their interaction. This regression technique is suitable for data sets that include non-independence between replicates of each sporophyte. For the variable D, the parameters are estimated as for a linear regression, whilst for G we estimated the parameters of a logistic regression due to the binomial character of the response variable. The models are as follows:

$$D=\beta_{0}+\beta_{1}∙\Delta t+\beta_{2}∙P+\beta_{3}∙\Delta t∙P$$

$$G=\frac{1}{1+e^{-(logit)}},where\;logit=\text{ln}\left(\frac{G}{1-G}\right)=\beta_{0}+\beta_{1}∙\Delta t+\beta_{2}∙P+\beta_{3}∙\Delta t∙P$$

So, the greater the logit, the greater the proportion of germination.

Significance in the interaction effect means that the differences showed in the responses, concerning the increase of temperature, also depended on population and vice versa. Consequently, it would allow us to analyze the influence of temperature increase separately for each population by means of additional GEE analyses. Regarding the population factor, we built a dummy variable P = 0 for the populations from the fragmented forest and P = 1 for the populations from the unfragmented forest.

To test the existence of interspecific variation, we built logistic and linear models using the relative reduction in germination and delay, respectively:

$$RRG=\frac{{\hat{G}}_{13}-{\hat{G}}_{17.5}}{{\hat{G}}_{13}};\;RRD=\frac{{\hat{D}}_{13}-{\hat{D}}_{17.5}}{{\hat{D}}_{13}}$$

and introducing the species as factor (Sp = 0, *A*. *dareoides*; Sp = 1, *A*. *trilobum*).

We used the estimated models to predict the germination behavior of the temperate epiphytic ferns under two scenarios of increasing temperatures [as defined by 51]: +2°C, which is slightly higher than the upper limit of RCP2.6, and +4.5°C, which is slightly lower than the upper limit of RCP8.5.

All the analyses were made with STATA v9.0, with a significance level of α = 0.05.

## Results

### Germination and vulnerability models of *A*. *dareoides* and *A*. *trilobum*

**Fig 2:**
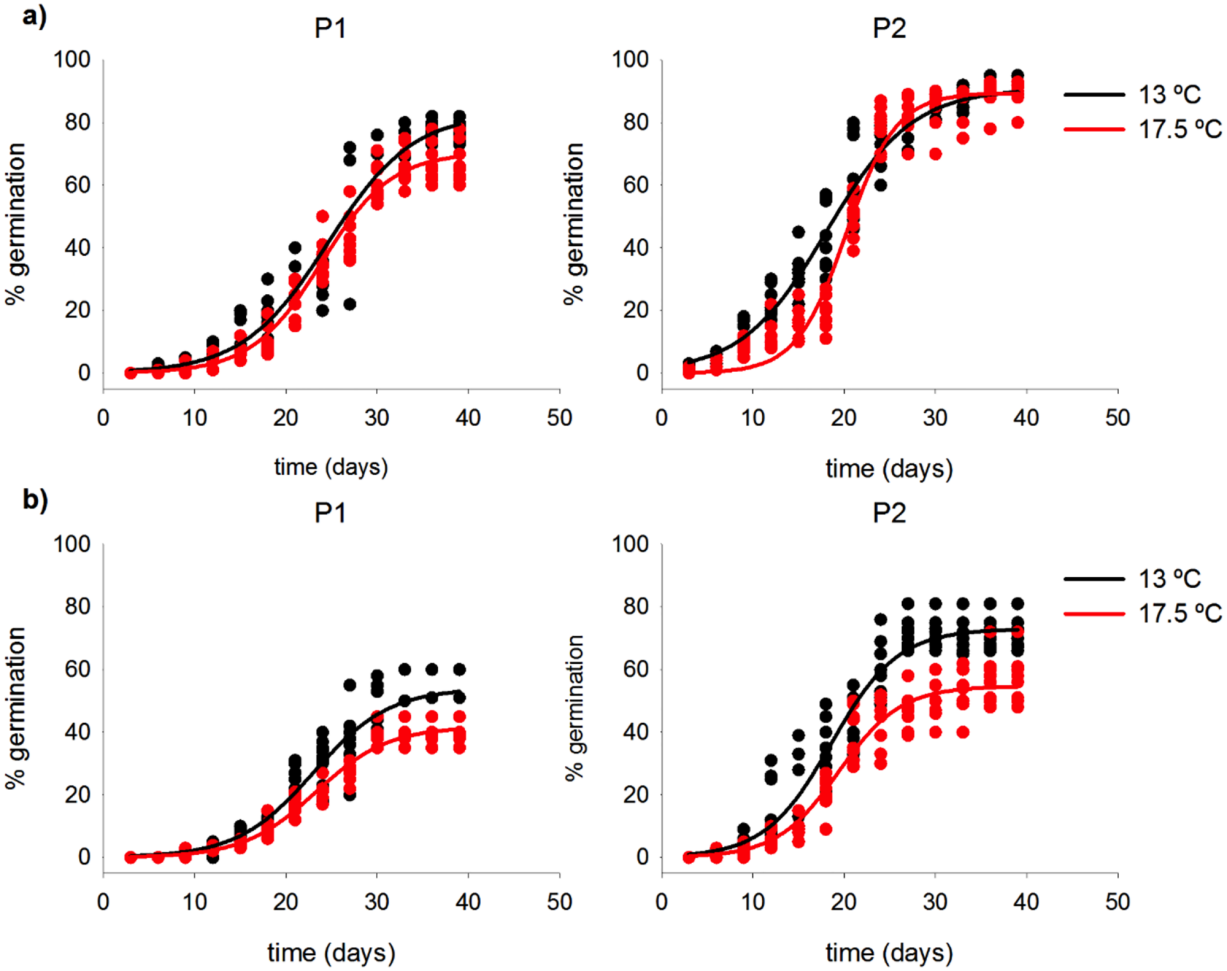
Fit of germination percentage over time to a logistic curve (points = raw data; lines = fit model). Each condition was fit separately, black and red points/lines indicate data and fit at 13°C and 17.5°C respectively. a) Data from *Asplenium dareoides*. b) Data from *Asplenium trilobum*. P1 indicates results for populations living in the fragmented forest, and P2 for populations living in the unfragmented forest.

The germination kinetics of both species fit significantly to a logistic sigmoid regression model (Fig 2). The response of the studied species to increasing temperatures and forest fragmentation was different depending on species, germination variable and population. All the estimated models and their statistical significance are shown in S1 Appendix.

**Fig 3:**
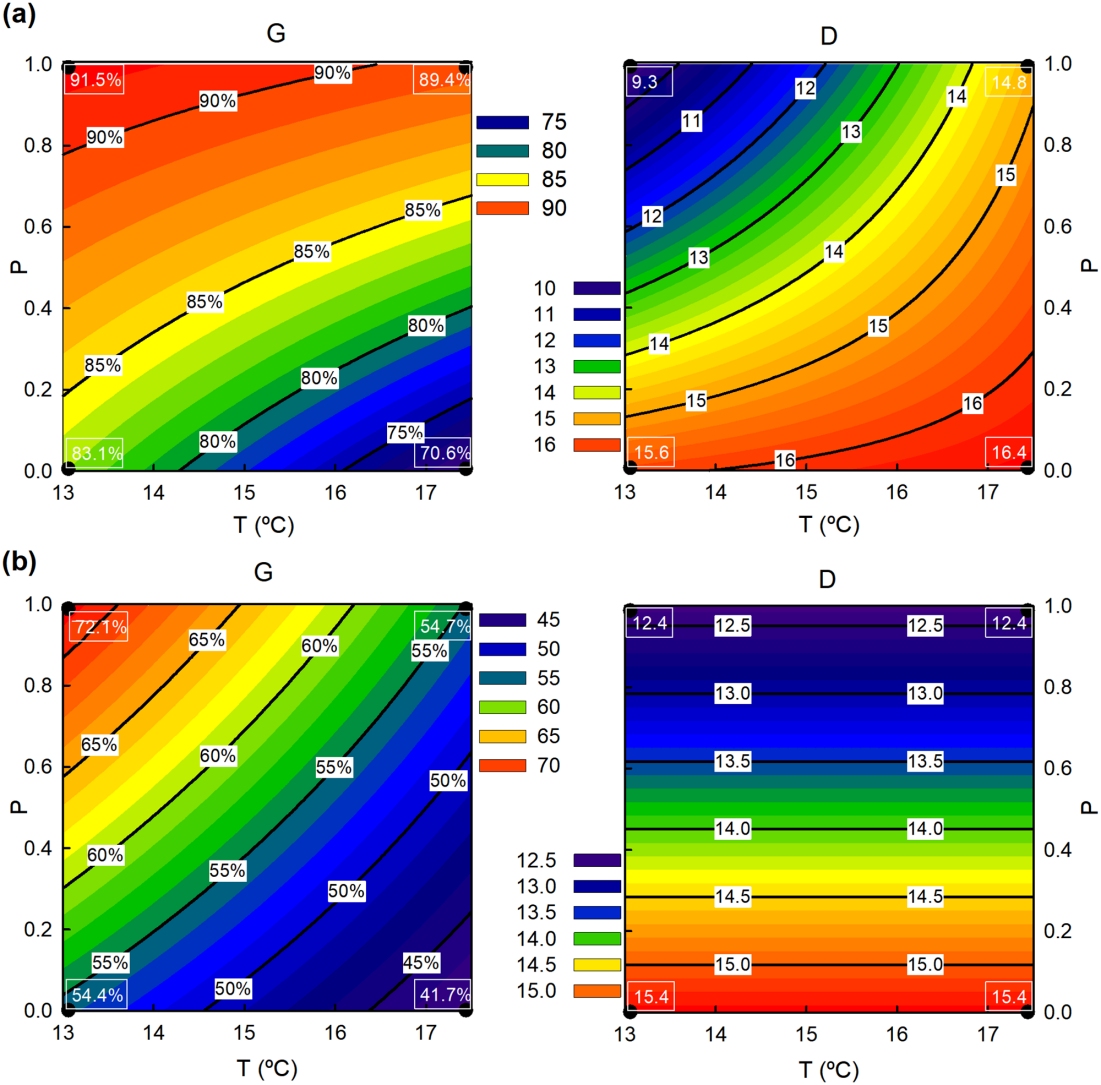
Relationships of germination parameters with population conditions. a) Data from *A*. *dareoides*. b) Data from *A*. *trilobum*. G = maximum germination proportion (showed as percentage), D = delay on germination, P = population conditions (from 0 = fragmented forest to 1 = unfragmented forest), T = temperature. Figures in the white boxes are the sampled actual data for each population-temperature pair.

The maximum germination of *A*. *dareoides* showed a strong correlation with the temperature increase, the population, and with the interaction between temperature and population. Following the model, we can state that, in general terms, the maximum germination of this species was reduced with increased temperature; also, regarding populations, individuals from P2 showed a stronger response (higher G in about 8.43%) when no temperature increase is considered (Fig 3a). Given the observed interaction between temperature and population, a further analysis was done to properly study the effect of the temperature on germination in each population. In this sense, we found an inverse correlation in P1, and in P2, but in this case with a smaller effect and without statistical significance. These models showed that the maximum germination reached by *A*. *dareoides* was reduced with temperature increase in both populations, but to different extent: the reduction was about 12.44% in the P1 for an increase of 4.5°C, but only about 2.05% in P2 (Fig 3a). The delay in the germination of *A*. *dareoides* exhibited also a significant correlation with population and the interaction between population and temperature, but not with the temperature alone. The estimated model indicates that the germination delay in *A*. *dareoides* generally increases with temperature. Also, if no increase of temperature is considered, individuals from P2 showed a germination delay about 6.6 days shorter than those from P1 (Fig 3a). Again, as the interaction between the two factors is significant, we studied the effect of the temperature in each population separately. From these models, we can state that the delay in the germination is increased to about 1.2 days per degree of increment in the temperature in P2, but temperature does not appear to significantly affect the delay in P1 (Fig 3a).

The germination of *A*. *trilobum* showed an inverse correlation with the increment of temperature, a direct correlation with the population, and an inverse correlation with the interaction between population and temperature. In this species, the increasing temperature is also reducing the germination proportion. As an interaction between population and temperature has been detected, we carry on a further analysis to study the influence of temperature over germination for each of the populations. Under an increase of 4.5°C the models estimate a reduction of 12.67% in germination for P1, and 17.39% for P2 (Fig 3b). For *A*. *trilobum*, a significant correlation was measured between delay and population but not with the temperature or with the interaction between the two factors. The estimated model indicates that, on the one hand, the delay seemed not to be affected by increased temperature, and on the other hand, the delay was significantly larger in P1 (in about 3 days) with respect to P2 (Fig 3b).

### Interspecific differences in the germination vulnerability

The resulting models (S2 Appendix) show a higher reduction in the maximum germination in *A*. *trilobum* than in *A*. *dareoides*. This difference is more noticeable in P2, where there is a relative reduction of 2.1% in *A*. *dareoides* but of 24.1% in *A*. *trilobum*, whereas in P1 the relative reduction is of 15.0% for *A*. *dareoides* and 23.2% for *A*. *trilobum*. Regarding the germination delay, there is a higher increment in *A*. *dareoides* than in *A*. *trilobum* for both populations. This difference is much higher in the unfragmented forest, where in the sample there is a change of 63% for *A*. *dareoides* but only of 20.4% for *A*. *trilobum*.

### Vulnerability prediction for several increased temperature scenarios

**Fig 4:**
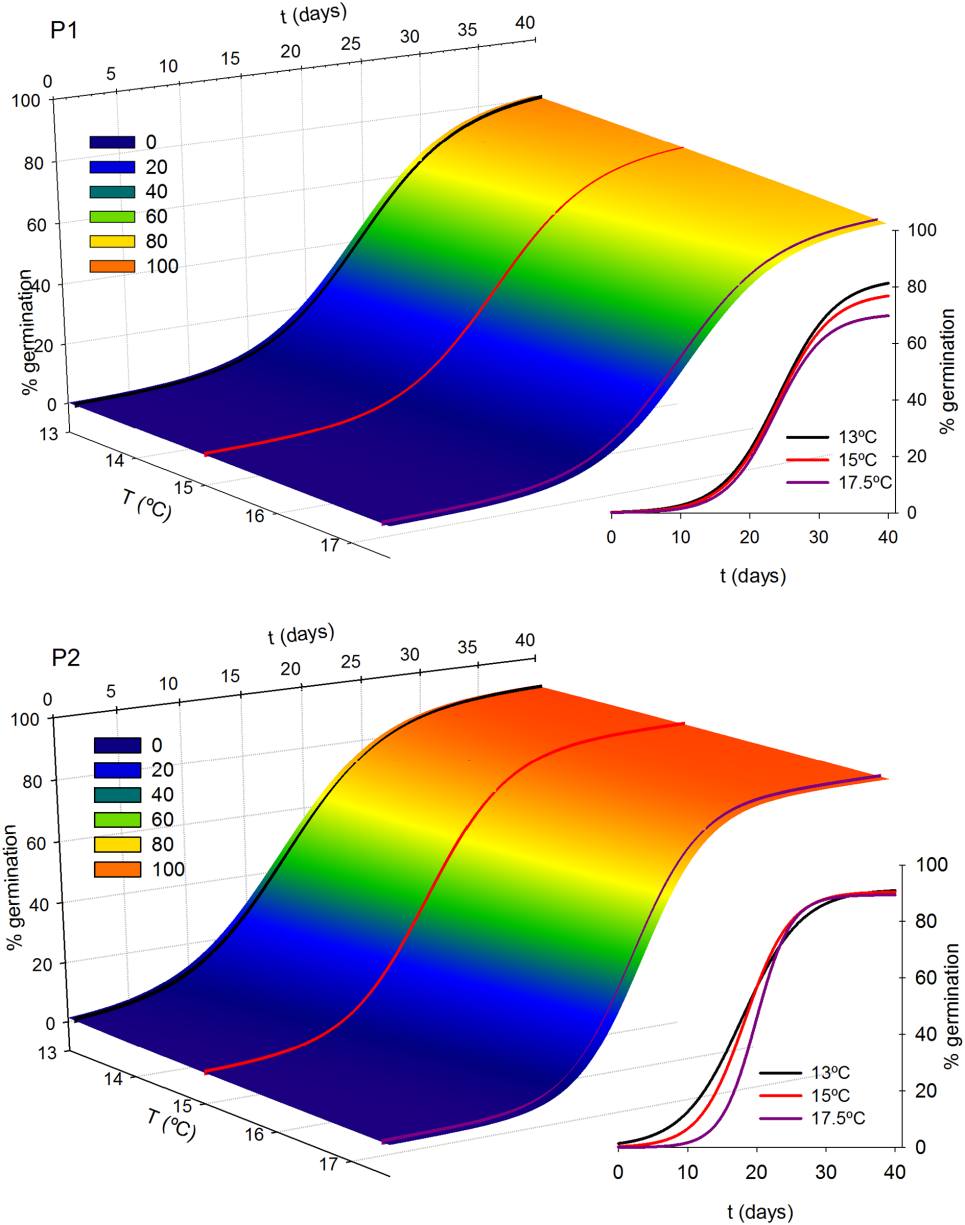
Germination curves of *A*. *dareoides* versus temperature for populations from the fragmented forest (P1) and populations from the unfragmented forest (P2). Inset, current temperatures (13°C), and estimations at +2°C and +4.5°C scenarios.

**Fig 5:**
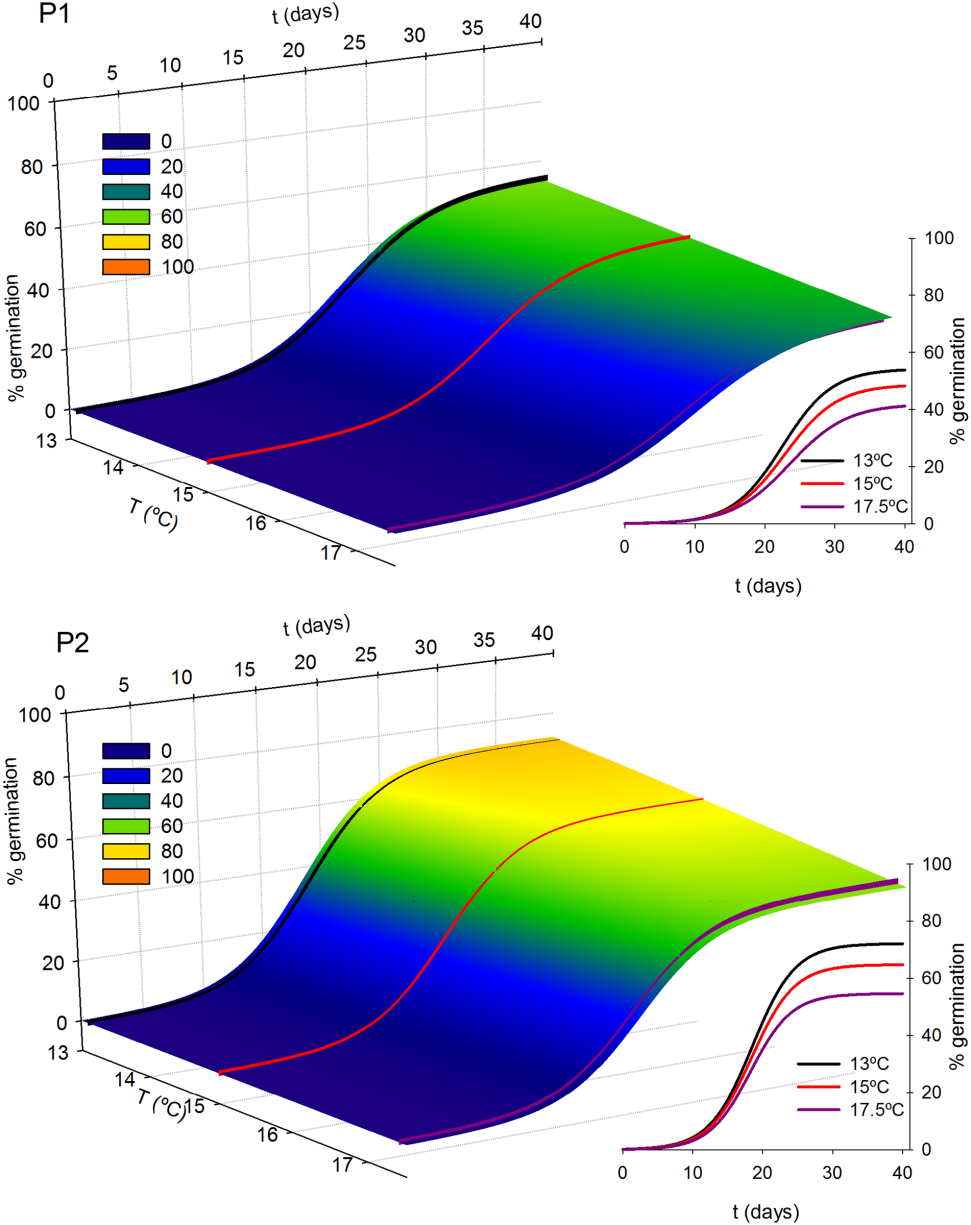
Germination curves of *A*. *trilobum* versus temperature for populations from the fragmented forest (P1) and populations from the unfragmented forest (P2). Inset, current temperatures (13°C), and estimations at +2°C and +4.5°C scenarios.

Under the RCP2.6 scenario of climate change (Table 1, Figs 4 and 5), the maximum percentage of germination is reduced in almost 5% in mean for all species and populations. This reduction is even more significant in P1. *Asplenium trilobum* seems to be more vulnerable than *A*. *dareoides*, showing more than a double reduction in the maximum percentage of germination. The effect of the increasing temperature over the germination delay is virtually nil (+0.68 days for all species and populations). Nevertheless, the effect is slightly higher on the individuals of P2. In this case, *A*. *dareoides* is more vulnerable than *A*. *trilobum*, as the former shows delay in germination and the latter shows no significative change in this parameter.

**Table 1:** Predicted germination values (maximum germination in %, and delay in the onset of germinations in days) compared between species and populations, reflecting differences between spores growing under actual temperatures and under the two scenarios of increasing temperatures considered.

		RCP2.6 (+2°C)			RCP8.5 (+4.5°C)		
		P1	P2	Mean	P1	P2	Mean
Δ Germ (%)	*A*. *dareoides*	-4.99	-0.81	-2.90	-12.44	-2.05	-7.24
	*A*. *trilobum*	-5.49	-7.32	-6.40	-12.67	-17.39	-15.03
	Mean	-5.24	-4.07	-4.66	-12.56	-9.72	-11.14
Δ Delay (days)	*A*. *dareoides*	+0.26	+2.46	+1.36	+0.58	+5.53	+3.05
	*A*. *trilobum*	0.0	0.0	0.0	0.0	0.0	0.0
	Mean	+0.13	+1.23	+0.68	+0.29	+2.76	+1.52

Under a more dramatic RCP8.5 scenario of increasing temperatures, the maximum germination is reduced by more than 11% considering all the species and populations. The reduction is much higher in P1. *Asplenium trilobum* is more vulnerable, suffering almost a double reduction in its germination percentage. Germination is delayed 1.52 days for all the species and populations, an effect more noticeable in P2. In this regard, *Asplenium dareoides* shows more vulnerability.

## Discussion

The logistic models estimated here agree with other sigmoid saturation models that have been reported for the germination of other fern species [52]. Our results suggest that increased temperature can reduce spore germination and alter germination kinetics on two temperate epiphytic ferns, a finding similar to long-term observations of the impacts of climate change on other plants [53], considering that most of the studies have focused on trees and crops. Climate change, even in the less hazardous scenario RCP2.6, will have a negative impact over temperate epiphytic fern populations. The impact is stronger in the RCP8.5 scenario, but the increase in temperature does not follow a linear pattern of increasing effects. The warmer temperatures seem to negatively impact germination percentages more than delay of germination. Many factors can impact local weather and microclimate, for example, changes in forest structure, the clearing of trees, and the fragmentation of forest due to changes in land use can influence environmental conditions critical for epiphytic species. The combined impacts of multiple stresses on plant physiology can exponentially impact species biology [21]. The loss of tropical and temperate primary forests is linked to the disappearance of moist-sensitive fern epiphytes [25, 40]. This could also happen in our case, as we have observed that well-structured non-fragmented forests seem to smooth the reduction of maximum germination or the delay in the onset of germination, due to increasing temperatures.

Not all species respond in the same way to climate change, as diverse biological and ecological capacities could result in differential vulnerability to environmental changes [8, 13, 42]. In our case, the two species investigated show a slightly different response to increasing temperatures: *Asplenium trilobum* seems to be more vulnerable in the percentage of germination and *A*. *dareoides* in the delay of the onset of germination. Thus, if climate change follows the predicted patterns (+2°C or +4.5°C), our models estimate a stronger depletion of populations of *A*. *trilobum* and a moderate reduction of *A*. *dareoides* in temperate humid forests, this reduction being much more significant in less structured habitats. In other words, *A*. *trilobum* seems a more mesic species than the more drought-resistant *A*. *dareoides*, whose populations could become preponderant with the climate change.

It is known that, from spore germination to sexual reproduction, there is a mortality rate in all stages of gametophyte development, variable according to species and habitat [33, 54, 55]. In this context, a minimum spore germination guarantees that, in the subsequent gametophytic populations, there are enough individuals to ensure, on one hand, new colonization events, and on the other hand, a proper amount of sexual intergametophytic contacts, to maintain genetic diversity and adaptive possibilities [43, 44, 56]. Thus, the observed phenomenon of germination reduction will have effect on population recruitment and dynamics: reduction in germinated spores in the elements of the forest canopy (e.g. host trunks) will result in fewer gametophytic individuals and decreased sexual reproduction for future populations. For the epiphytic communities even a moderate environmental stress could result in a fecundity decrease with important consequences for the community [17], irrespective of the presumable maintenance of a viable spore/seed bank and the possible existence of clonal survival strategies [57]. It is quite difficult to precisely assess from our data the exact amount of recruitment reduction and population shrinkage under increased temperature and forest fragmentation (i.e. which is the significance of, for example, a 10% reduction of spore germination in terms of gametophytic population): this issue needs an experimental approach which was not included in our current objectives, but will be addressed in the future. Anyway, in the long term, and if changes in environmental conditions persist at least at current rates, temperate fern epipythic populations and communities will be negatively affected.

### Conservation implications

Our study draws attention to the role of ferns in epiphytic communities of temperate forests, an understudied group of organisms with an important function in their ecosystems. The results of our experiments inform about the vulnerability of these plants faced to changes drifted by human activities. This fact agrees with previous research in the sense of the need to increase the conservation efforts to maintain plant epiphytic populations [23, 58].

Since it has been demonstrated that the two environmental problems studied here (global warming and deforestation) affect the vulnerability of epiphytic temperate ferns, both should be considered from a conservationist perspective. However, without forgetting the absolutely necessary efforts to reduce global warming sources, in the field of epiphytic plant conservation, it may be more effective to insist on the need to maintain a minimum and sufficient forest structure. Indeed, it is reasonable to think that epiphytes could acclimatize their metabolism to an environmental temperature change, but they certainly cannot live without their host trees [21].

Therefore, the main conservationist implication of our study is directed towards the conservation and restoration policy of South American forests. Thus, we agree with previous authors on the need to reinforce the efforts that have already been implemented [46], but here drawing attention to another of the elements of these biological systems. The conservation of forest fragments, even any small patch, has been already pointed as important strategy for epiphytic plants [59, 60]. Although many of these directions have been done studying epiphytic angiosperms from tropical and subtropical environments, it is obvious that similar conservational efforts would have benefits for temperate epiphytic ferns. In addition to a classical approach regarding forest patches and corridors as conservational elements, the practice of artificial translocation [61] should also be considered to improve conservation of epiphytic fern communities.

From a methodological point of view, the importance of epiphytes as models in a conservationist context has been previously signified [21, 62]. Here, we propose a new model to detect shift in temperate epiphytic fern germination kinetics (and consequently, to detect variations in community composition) due to human-drifted environmental changes. An important feature of our model is the simplicity and usefulness of the variables used in relation to spore germination. Thus, these model and methodology could be easily applied to dynamically monitor the status of ecosystems and to tack changes in communities, allowing the quick prediction of possible future scenarios, which is a crucial issue in biodiversity conservation planning, particularly for epiphytic communities [63].

Finally, another interesting aspect of the method here proposed is its ease of being applied to other spore-dispersing organisms, as bryophytes, and also to cryptogamic terrestrial communities.

## Supporting information

S1 Appendix Estimated vulnerability models of germination fitness for *A*. *dareoides* and *A*. *tribolum*. (PDF)

S2 Appendix Models related to the interspecific differences in germination fitness between *A*. *dareoides* and *A*. *tribolum*. (PDF)
