# Supplementary material for: Germination fitness of two temperate epiphytic ferns shifts under increasing temperatures and forest fragmentation
Source: PLoS One. 2018 May 11;13(5):e0197110. doi: 10.1371/journal.pone.0197110 (PMC5947888; doi:10.1371/journal.pone.0197110)
Supplement: S1 Appendix — (PDF) [file pone.0197110.s001.pdf]

S1 Appendix

Estimated vulnerability models of germination fitness for *A. dareoides* and *A. tribolum*

1. *Asplenium dareoides*

A) Germination percentage

General: logit (G)= 1.59 – 0.16·ΔT + 0.78·P + 0.11·ΔT·P ; $p<0.001$

Population 1: logit (G)= 1.59 – 0.16·ΔT ; $p<0.001$

Population 2: logit (G)= 2.37 – 0.05·ΔT ; $p = 0.135$

B) Delay in germination

General: D= 15.78 + 0.13·ΔT – 6.60·P + 1.10·ΔT·P ; $p<0.001$

Population 1: D= 15.78 + 0.13·ΔT ; $p = 0.473$

Population 2: D= 9.26 + 1.23·ΔT ; $p < 0.001$

2. *Asplenium trilobum*

A) Germination percentage

General: logit (G)= 0.17 – 0.11·ΔT + 0.78·P – 0.06·ΔT·P ; $p<0.001$

Population 1: logit (G)= 0.17 – 0.11·ΔT ; $p<0.001$

Population 2: logit (G)= 0.95 – 0.17·ΔT ; $p=0.135$

B) Delay in germination

General: D= 15.35 – 3.00·P ; $p<0.01$
