# Supplementary material for: Germination fitness of two temperate epiphytic ferns shifts under increasing temperatures and forest fragmentation
Source: PLoS One. 2018 May 11;13(5):e0197110. doi: 10.1371/journal.pone.0197110 (PMC5947888; doi:10.1371/journal.pone.0197110)
Supplement: S2 Appendix — (PDF) [file pone.0197110.s002.pdf]

S2 Appendix

Models related to the interspecific differences in germination fitness between *A. dareoides* and *A. tribolum*

Population 1

logit (RRG)= $-1.74 + 0.53 \cdot Sp$; $p<0.767$

RRD= $0.14 + 0.06 \cdot Sp$; $p = 0.093$

Population 2

logit (RRG)= $-3.80 + 2.66 \cdot Sp$; $p<0.001$

RDD= $-0.63 + 0.42 \cdot Sp$; $p<0.001$
